# Haemoptysis and bronchial congestion due to pulmonary vein stenosis after maze procedure

**DOI:** 10.1002/rcr2.467

**Published:** 2019-08-13

**Authors:** Takayuki Nakaizumi, Kei Nakamura, Kentaro Nakamura, Masanobu Ishigaki, Haruki Taniguchi, Koichiro Kajiura

**Affiliations:** ^1^ Department of Emergency and Critical Care Medicine Urasoe General Hospital Okinawa Japan; ^2^ Department of Thoracic Center Urasoe General Hospital Okinawa Japan; ^3^ Department of Cardiovascular Medicine Urasoe General Hospital Okinawa Japan

**Keywords:** Bronchial congestion, haemoptysis, maze procedure, pulmonary vein stenosis

## Abstract

Pulmonary vein stenosis (PVS) is a rare disease that can cause haemoptysis. Acquired PVS is indicated as a complication associated with cardiac catheter intervention; however, the maze procedure has not been reported to induce PVS. Here, we describe the diagnosis and treatment strategy for the first case of PVS with haemoptysis due to the maze procedure. A 56‐year‐old man who underwent the maze procedure seven years previously was referred for repeated haemoptysis. Contrast‐enhanced computed tomography (CT) revealed complete occlusion of the left superior pulmonary vein. Bronchoscopy revealed localized bronchial congestion and varices. He was diagnosed with PVS due to the maze procedure, and he underwent catheter‐balloon angioplasty. After treatment, haemoptysis disappeared and bronchial congestion and varices improved. History of cardiac ablation (surgical or catheter intervention) and localized bronchial congestion findings might facilitate the accurate diagnosis of PVS with haemoptysis. Catheter‐balloon angioplasty is a minimally invasive treatment for PVS.

## Introduction

Pulmonary vein stenosis (PVS) is a rare congenital or acquired disease. Acquired PVS is a complication of cardiopulmonary surgery or catheter intervention 1. In adults, it is one of the most serious complications caused by radiofrequency catheter ablation for atrial fibrillation (AF). PVS is defined as stenosis of >50% of the pulmonary vein diameter 2. PVS might induce pulmonary hypertension, exertional dyspnoea, chest pain, right ventricular heart failure, pulmonary oedema, and intermittent haemoptysis. Symptomatic PVS can be treated via catheter‐balloon angioplasty, surgical angioplasty, stent implantation, or lung resection 1.

The maze procedure is aimed at pulmonary venous isolation for AF surgical ablation. Here, we describe the diagnosis and treatment strategy for the first case of PVS with haemoptysis due to the maze procedure.

## Case Report

**Figure 1:**
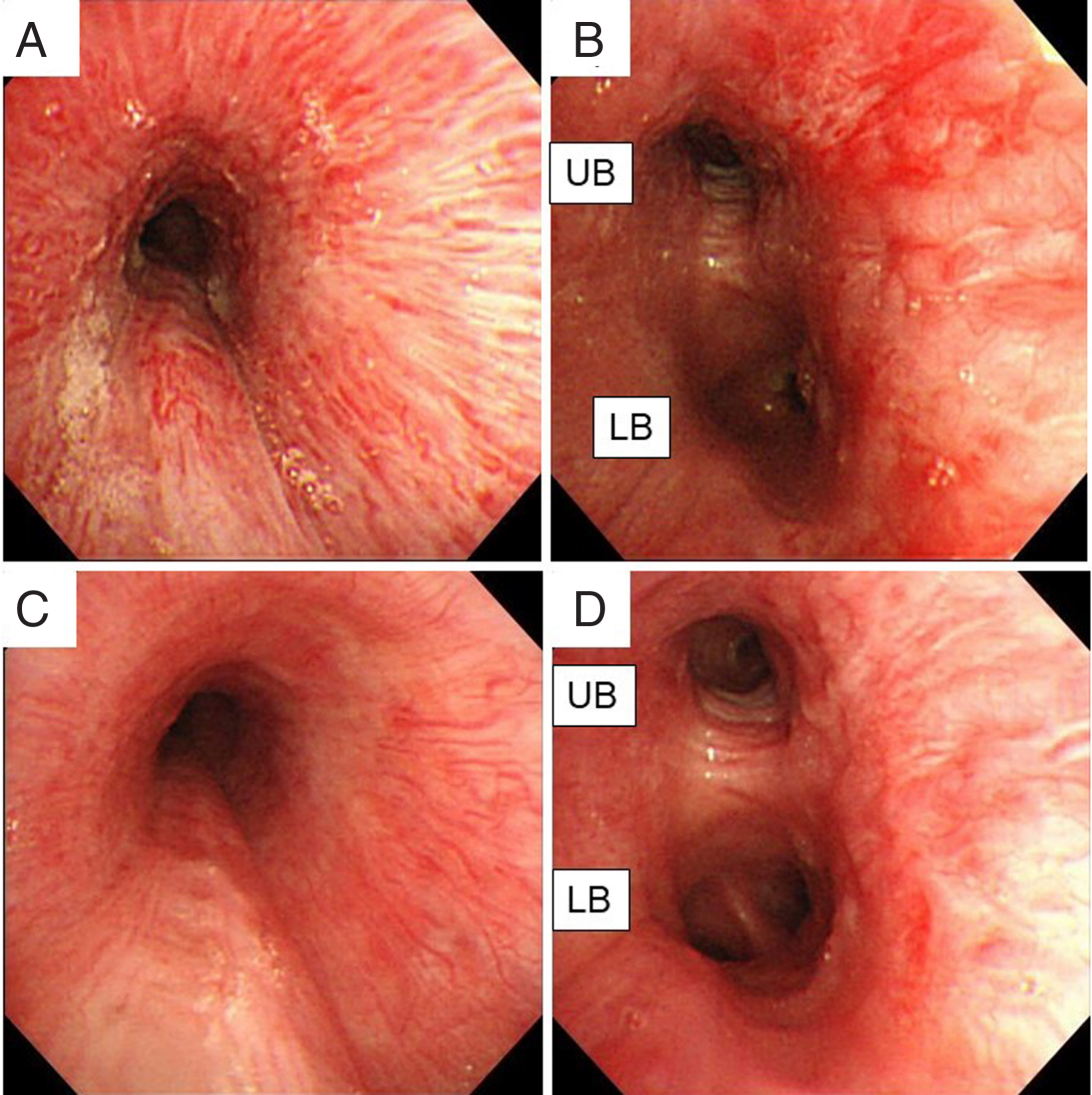
Bronchoscopic findings showing bronchial congestion and varices before catheter treatment (A, left main bronchus view; B, second carina view). Bronchoscopic findings showing improved bronchial congestion and varices after catheter treatment (C, left main bronchus view; D, second carina view). UB, upper bronchus; LB, lower bronchus.

**Figure 2:**
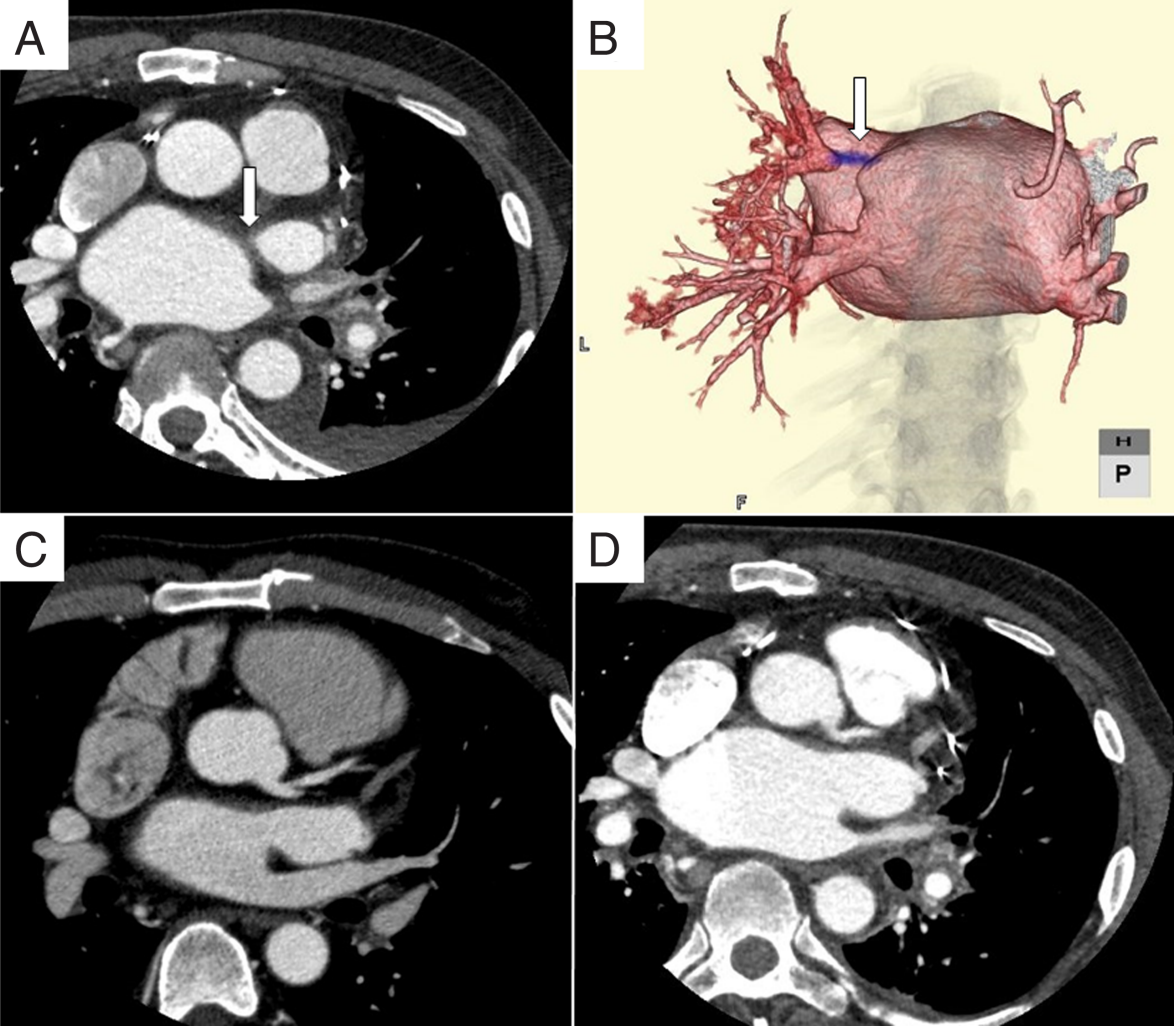
(A) Contrast‐enhanced computed tomography (CT) showing left superior pulmonary vein stenosis (PVS) (arrow). (B) A reconstructed posterior view contrast‐enhanced CT image of the left superior PVS (arrow). (C) Contrast‐enhanced CT showing that the left superior pulmonary vein is intact before the maze procedure. (D) Contrast‐enhanced CT showing the improvement in left superior PVS after catheter‐balloon angioplasty.

A 56‐year‐old man was referred to our hospital with massive haemoptysis. He had undergone tricuspid valve replacement (with a mechanical valve) owing to incompetence of the tricuspid valve with right heart failure and surgical ablation for AF (maze procedure) at another hospital seven years previously, and he was on warfarin. He complained of repeated haemoptysis after the cardiac surgery. In addition to clotting, bronchoscopy revealed local congestion and oedema in the lumen of the left main bronchus (Fig. 1A,B). Warfarin was discontinued and treatment with haemostatic drugs was initiated, as bronchoscopic biopsy showed no aetiological evidence for haemoptysis. Despite several bronchial arterial embolization procedures, recurrent haemoptysis was noted. A physical examination revealed the following: blood pressure, 185/83 mmHg; heart rate, 80 beats/min; respiratory rate, 30 breaths/min; and O_2_ saturation, 87% under room air. Because of the risk of airway obstruction, we performed immediate intubation. Bronchoscopy revealed massive bleeding and clotting in the left main bronchus. We performed one‐lung ventilation with a bronchial blocker. Contrast‐enhanced computed tomography (CT) revealed bilateral consolidation due to bronchial bleeding and left superior PVS (Fig. 2A,B). In addition, we confirmed that the left superior pulmonary vein was intact on CT before the maze procedure (Fig. 2C). PVS, haemoptysis, and bronchial congestion were present after the maze procedure. Therefore, we hypothesized that left superior PVS occurred as a complication of the maze procedure, and it involved bronchial congestion and repeated haemoptysis. Warfarin treatment was important for this patient after mechanical valve replacement, and PVS had to be improved to stop haemoptysis. Initially, astriction via a bronchial balloon blocker helped stop haemoptysis, and extubation could shortly be performed. We then performed transcatheter balloon dilatation for the left superior PVS via the Brockenbrough method. There was no haemoptysis after this procedure, even with warfarin administration. Follow‐up contrast‐enhanced CT revealed patency of the left superior pulmonary vein (Fig. 2D). The patient was discharged without any complications. At the six‐month follow‐up, no episode of haemoptysis had occurred and bronchial congestion had improved (Fig. 1C,D).

## Discussion

Symptomatic acquired PVS in adults is rare and has been reported as an iatrogenic complication of cardiac catheter intervention 1. However, there have been few cases involving acquired PVS due to the maze procedure in adults. Therefore, it was difficult to make an accurate diagnosis in the present case. Because the patient had undergone surgical ablation, we kept PVS in mind in the differential diagnosis of haemoptysis. Moreover, the bronchoscopic findings of localized bronchial congestion were helpful for diagnosing PVS 3.

As a possible cause of the acquired PVS after the maze procedure in the present case, part of the pulmonary venous isolation might have been closed slightly more towards the peripheral side than is usual in the maze procedure. However, this possibility could not be confirmed owing to unavailability of the surgical record as the patient had undergone the surgery at another hospital seven years previously.

Symptomatic PVS can be treated with catheter‐balloon angioplasty, surgical angioplasty, stent implantation, or lobe resection 2. In the present case, catheter‐balloon angioplasty was chosen as the least invasive procedure. However, there was a high risk of perforation because of complete occlusion of the left superior pulmonary vein and altered organization of the vessel tissue due to the seven‐year period after the maze procedure. Thus, we prepared for drainage of the pericardial sac and emergency operation. Restenosis might occur in more than 50% of patients within one year 1. Thus, careful follow‐up after balloon angioplasty is necessary. In our patient, if restenosis occurs in the pulmonary vein after catheter‐balloon angioplasty, we plan to perform stent implantation. We considered that stent implantation was unnecessary at the initial catheter treatment, as the patient had taken warfarin for mechanical valve replacement. Surgical angioplasty and lobar resection were considered in the surgical approach. However, we were worried about adhesions, bleeding, and surgical complications associated with the previous tricuspid valve replacement in the case of surgical angioplasty. Additionally, lung function was decreased in a part of the left superior lobe, which would be problematic in the case of lobar resection, although lobar resection can be performed less invasively by video‐assisted thoracic surgery. Therefore, catheter‐balloon angioplasty was chosen as the least invasive method in the present case. It is important to select the most appropriate treatment for each patient with PVS.

### Disclosure Statement

Appropriate written informed consent was obtained for the publication of this case report and accompanying images.
